# Association between omentin-1 and major cardiovascular events after lower extremity endovascular revascularization in diabetic patients: a prospective cohort study

**DOI:** 10.1186/s12933-020-01151-z

**Published:** 2020-10-07

**Authors:** Federico Biscetti, Elisabetta Nardella, Maria Margherita Rando, Andrea Leonardo Cecchini, Flavia Angelini, Alessandro Cina, Roberto Iezzi, Marco Filipponi, Angelo Santoliquido, Dario Pitocco, Raffaele Landolfi, Andrea Flex

**Affiliations:** 1 Fondazione Policlinico Universitario A. Gemelli IRCCS, Rome, Italy; 2 Internal Medicine and Vascular Diseases Unit, Rome, Italy; 3 Department of Translational Medicine and Surgery, Laboratory of Vascular Biology and Genetics, Rome, Italy; 4 Department of Radiology, Rome, Italy; 5 Ospedale San Giovanni Battista—ACISMOM, Rome, Italy; 6 Università Cattolica del Sacro Cuore, Rome, Italy; 7 Angiology Unit, Rome, Italy; 8 Diabetology Unit, Rome, Italy; 9 Department of Internal Medicine, Fondazione Policlinico Universitario A. Gemelli IRCCS, Catholic University School of Medicine, Largo Francesco Vito, 1, 00168 Rome, Italy

**Keywords:** Type 2 diabetes mellitus, Chronic limb-threatening ischemia, Major adverse limb events, Outcome prediction, Omentin-1

## Abstract

**Background:**

Cardiovascular complications represent the major cause of morbidity and mortality of type 2 diabetes mellitus (T2DM) patients. In particular, peripheral artery disease (PAD) represents a frequent T2DM vascular complication and a risk factor for the development of major adverse cardiovascular events (MACE). Among adipokines, omentin-1 serum levels are reduced in T2DM patients with PAD and are inversely related to disease severity.

**Objective:**

To study the relationship between omentin-1 levels, at baseline, with outcomes after endovascular procedures in T2DM patients with PAD and chronic limb-threatening ischemia (CLTI).

**Research design and methods:**

We enrolled for our prospective non-randomized study, 207 T2DM patients with PAD and CLTI, requiring revascularization. Omentin-1 serum levels were collected before revascularization and patients incidence outcomes were evaluated at 1, 3, 6 and 12 months.

**Results:**

Omentin-1 was reduced in patients with more severe disease (27.24 ± 4.83 *vs* 30.82 ± 5.48 ng/mL, p < 0.001). Overall, 84 MACE and 96 major adverse limb events (MALE) occurred during the 12-month follow-up. We observed that omentin-1 levels were lower in patients with MACE (26.02 ± 4.05 *vs* 31.33 ± 5.29 ng/mL, p < 0.001) and MALE (26.67 ± 4.21 *vs* 31.34 ± 5.54 ng/mL, p < 0.001). The association between omentin-1, MACE and MALE remained significant after adjusting for major risk factors in a multivariate analysis. Receiver operating characteristics (ROC) curve using omentin-1 levels predicted incidence events (area under the curve = 0.80).

**Conclusions:**

We demonstrated that reduced omentin-1 levels, at baseline, are related with worse vascular outcomes in T2DM patients with PAD and CLTI undergoing an endovascular procedure.

## Background

Peripheral artery disease (PAD) represents one of the most frequent vascular complications of type 2 diabetes mellitus (T2DM) [1]. PAD is also an independent risk factor for further cardiovascular complications in T2DM patients [2]. Treatment of PAD includes the reduction of modifiable risk factors, such as smoking and obesity and the improvement of medical therapy for diabetes, high blood pressure and dyslipidemia [3]. Despite the best medical cures, several patients aggravate and experience chronic limb-threatening ischemia (CLTI), lower limb ulcers and amputations [2]. In the case of CLTI, lower limb revascularization (LER) is suggested. Among LER approaches, endovascular revascularization is an effective option [4]. Notwithstanding vascular centers’ expertise, numerous treated patients experience remarkably different outcomes [5]. In fact, after LER, a considerable percentage of individuals develop major adverse cardiovascular events (MACE) or major adverse limb events (MALE) [4]. For this reason, biomarkers are necessary to stratify the risk of these patients and to design a more effective follow-up.

Among possible factors involved in atherosclerosis and vascular complications of T2DM, adipokines have been discussed in numerous reports and significant evidence is available [6]. Among the adipokines, omentin-1 is a protein closely associated to obesity, visceral fat and diabetes [7–11]. In particular, omentin-1 levels are negatively related to insulin resistance [9] and coronary artery disease (CAD) [12–14]. Moreover, omentin-1 seems to have beneficial effects in obese patients at higher cardiovascular risk [15]. Furthermore, we recently demonstrated that omentin-1 levels are inversely correlated to the presence of PAD and disease severity in T2DM patients [16].

Given the existing data, we postulate that the occurrence of cardiovascular events after LER may dependent on basal omentin-1 levels.

The purpose of this study is to assess the relationship between omentin-1 levels and vascular outcomes, in particular MACE and MALE, after endovascular revascularization in T2DM patients with PAD and CLTI.

## Research design and methods

### Study design

This study was designed as a prospective non-randomized study to verify the relationship between omentin-1 levels and the incidence of MACE and MALE after LER performed in T2DM patients with PAD and CLTI. The study was approved by the Ethics Committee of the *Fondazione Policlinico Universitario A. Gemelli IRCCS* and adhered to the principles of the Declaration of Helsinki. All the individuals agreed to participate in the study and provided informed consent.

### Study population and clinical assessment

Overall, 207 T2DM patients with PAD and CLTI below-the-knee were included and followed for the entire duration of the study. Patients were consecutively enrolled during a period between 30/05/2018 and 15/04/2019 at the Fondazione Policlinico Universitario A. Gemelli IRCCS, Rome, Italy. Inclusion criteria were age of 18 years or older, T2DM diagnosis [16] at least 1 year prior to the study, ankle/brachial index (ABI) lower than 0.80, peripheral artery stenosis greater than 50% documented by duplex ultrasound (US), presence of PAD at Rutherford category 4 or 5, presence of CLTI requiring endovascular treatment, no infections at present or in the previous month. In case of diabetic foot ulcers, additional criteria would be no local signs of infection and no need for antibiotic therapy. The wound, ischemia, foot infection (WIfI) classification system was used to stratify T2DM patients with foot ulcers. Radiological examination was performed, according to clinical judgment, to exclude osteomyelitis. Exclusion criteria were lower limb endovascular treatment or previous lower limb bypass surgery within the past 3 months, diabetic peripheral neuropathy, systemic steroid use or a prior history of use in the previous month, pregnancy, active cancer, life expectancy < 12 months, known liver disease with a functional status of B or above according to the Child–Pugh classification, congenital or acquired thrombophilia and active autoimmune disease [17].

All subjects were studied to rule out the presence of diabetic peripheral neuropathy, as previously described [18, 19]. Briefly, an assessment of vibration perception threshold was performed with a biothesiometer. All diabetic patients received a definite diagnosis of peripheral neuropathy with a Neuropathy Disability Score > 5 and a pathological conduction velocity. Autonomic neuropathy was diagnosed according to the standardized procedure of Ewing and Clarke, including four cardiovascular autonomic tests [20].

For all patients, additional clinical data was collected, including age, body mass index (BMI), history of cardiovascular diseases (CAD), cerebrovascular disease (CVD), hypertension, hypercholesterolemia, smoking, renal failure [defined as an estimated glomerular filtration rate (eGRF) < 60 mL/min]. All patients underwent a complete US peripheral vascular evaluation, and PAD was defined according to the criteria established by the ad hoc Committee on Reporting Standards of the Society for Vascular Surgery and the International Society for Cardiovascular Surgery [21]. In subjects with an ABI of 1.40 or more (uncertain arterial calcification), US evaluation was performed to assess significant stenosis of the peripheral arteries [22].

At the time of enrollment, all patients were on statin treatment, and they continued therapy after LER, aiming at low-density lipoprotein cholesterol (LDL-C) levels less than 70 mg/dl.

Patients were on single antiplatelet therapy, which was modified to dual antiplatelet therapy after LER for the following month.

### Revascularization treatment and follow-up

Angiography of the lower limb arteries, balloon angioplasty and, if indicated, stenting were performed as previously described [23]. The procedure was successful if the residual arterial stenosis was less than 30% [24]. No major complications, defined according to the definitions of the Society of Interventional Radiology [24], were observed. Of the 224 patients who underwent LER, 17 (7.6%) had a poor primary outcome and were excluded from the study follow-up.

For the follow-up, patients were evaluated 1, 3, 6 and 12 months after the LER to assess incidence of MACE and MALE. MACE were defined as composite of myocardial infarction, stroke and cardiovascular death. MALE were defined as composite of acute limb ischemia, major vascular amputations, limb-threatening ischemia leading to urgent revascularization.

### Blood sampling procedures and biochemical assays

Blood sampling of patients was performed at baseline, just before the LER, after an overnight fast. Fasting glucose, serum creatinine, total cholesterol, LDL-C, triglycerides, and glycated hemoglobin, were determined. Renal function was calculated using eGFR, which was performed using the modification of diet in renal disease formula. Serum was prepared by centrifugation of blood samples, which was stored at − 80 °C until assayed. Serum omentin-1 levels were determined by a commercially available ELISA kit (E-EL-H2028, Elabscience) according to its protocol. The intra- and inter-assay coefficients of variation were 3.5 and 10.5%, respectively. The sensitivity, defined as the mean ± 3 SD of the 0 standard, was calculated to be 0.15 pmol/mL. For each patient, the serum levels were measured twice, and the results were averaged.

### Statistical analysis

Data was summarized as means (standard deviations) for continuous variables and counts (percentages) for categorical variables. Demographic and clinical data of the groups were compared using Chi-square and t-test. Omentin-1 serum levels were compared with Mann–Whitney, Kruskal–Wallis and Dunn’s Multiple Comparison, when appropriate. A log transformation was applied to the not normally distributed variables prior to performing further analysis. A multivariate stepwise logistic regression analysis was performed, adjusted for traditional risk factors and omentin-1 levels. The area under the receiver operating characteristics (ROC) curve was calculated to test the predictive discrimination of MACE. The freedom from MACE according to the quartiles of serum omentin-1 was estimated using the Kaplan–Meier method and compared using the Log-Rank test. All analyses were performed using STATA version 14.0 for MacOS (Statistics/Data Analysis, Stata Corporation, College Station, TX, USA) and SPSS version 25.0 for MacOS (IBM Corporation, Armonk, NY, USA). Statistical significance was established at p < 0.05.

## Results

### Demographic and clinical characteristics

**Fig. 1:**
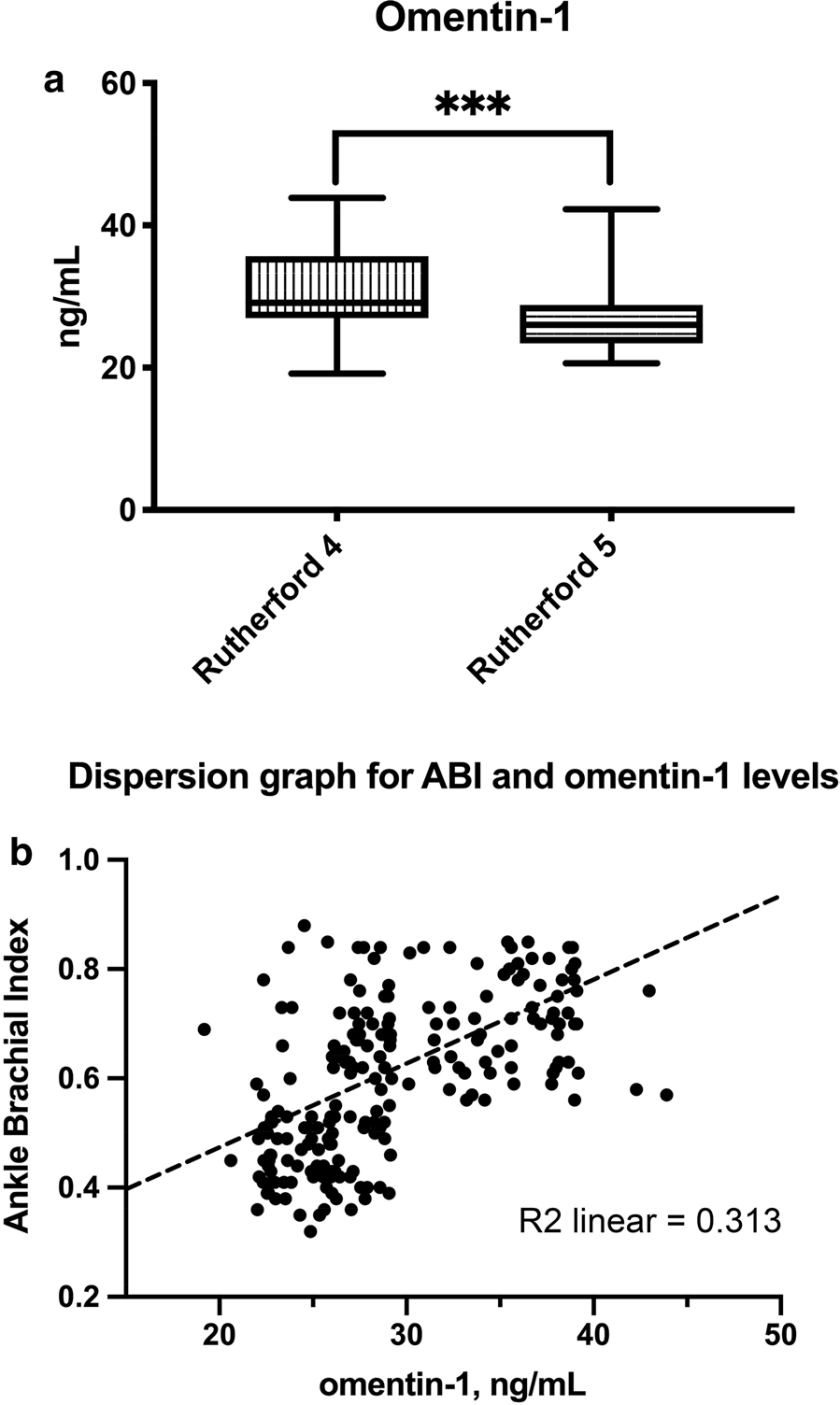
**a** Omentin-1 levels according to PAD severity. On the box plots, central lines represent the median, the length of the box represents the interquartile range and the lines extend to minimum and maximum values. ***P < 0.001. **b** Dispersion graph showing the correlation between Ankle Brachial Index (ABI) and omentin-1 serum levels.

Overall, 207 T2DM patients with PAD and CLTI were followed for the entire duration of the study. The mean age (SD) of patients was 75 (9.0) years. Of these, 144 (69.6%) were males. Patients had in average 11.4 (0.8) years T2DM. Complete clinical features are shown in Table 1. Patients with more severe PAD had lower serum omentin-1 levels than those with less severe PAD (27.24 ± 4.83 vs 30.82 ± 5.47 ng/mL, p < 0.001) (Fig. 1a). In addition, a strong positive correlation between omentin-1 levels and ABI were noted (Fig. 1b).

**Table 1:** Demographic characteristics of the study cohort at baseline

Number of patients	207
Age, years (SD)	75.0 (9.0)
Men, n (%)	144 (69.6)
Women, n (%)	63 (30.4)
Diabetes duration, years (SD)	11.4 (0.8)
BMI, kg/m^2^ (SD)	26.7 (0.6)
Oral antidiabetic agents, n (%)	92 (44.4)
Insulin, n (%)	152 (73.4)
High blood pressure, n (%)	144 (69.6)
Hypercholesterolemia, n (%)	113 (54.6)
Smoking status	
Never smoked, n (%)	57 (27.5)
Past smoker, n (%)	23 (11.1)
Current smoker, n (%)	127 (61.4)
ABI (SD)	0.39 (0.07)
Rutherford staging	
Stage 4, n (%)	112 (54.1)
Stage 5, n (%)	95 (45.9)
WIfI classification	
WIfI 010, n (%)	63 (30.4)
WIfI 020, n (%)	53 (25.6)
WIfI 110, n (%)	49 (23.7)
WIfI 120, n (%)	42 (20.3)
Previous coronary artery disease, n (%)	95 (45.9)
Previous cerebrovascular disease, n (%)	101 (48.8)
Total cholesterol, mg/dL (SD)	219.3 (22.3)
LDL cholesterol, mg/dL (SD)	112.7 (15.5)
Triglycerides, mg/dL (SD)	172.4 (8.5)
Glucose, mg/dL (SD)	128.0 (9.7)
HbA1C, % (SD)	8.9 (0.7)
eGFR, mL/min/1.73 m^2^ (SD)	72.8 (10.6)
Omentin-1, ng/mL (SD)	29.2 (5.4)

Data are reported as means (standard deviation) for continuous variables and numbers (percentages) for categorical variables. *BMI* Body Mass Index, *ABI* Ankle Brachial Index, *WIfI* Wound, ischemia, foot infection, *LDL* low-density lipoprotein, *eGFR* estimated glomerular filtration rate

### Serum omentin-1 and risk of MACE at 12 months

**Fig. 2:**
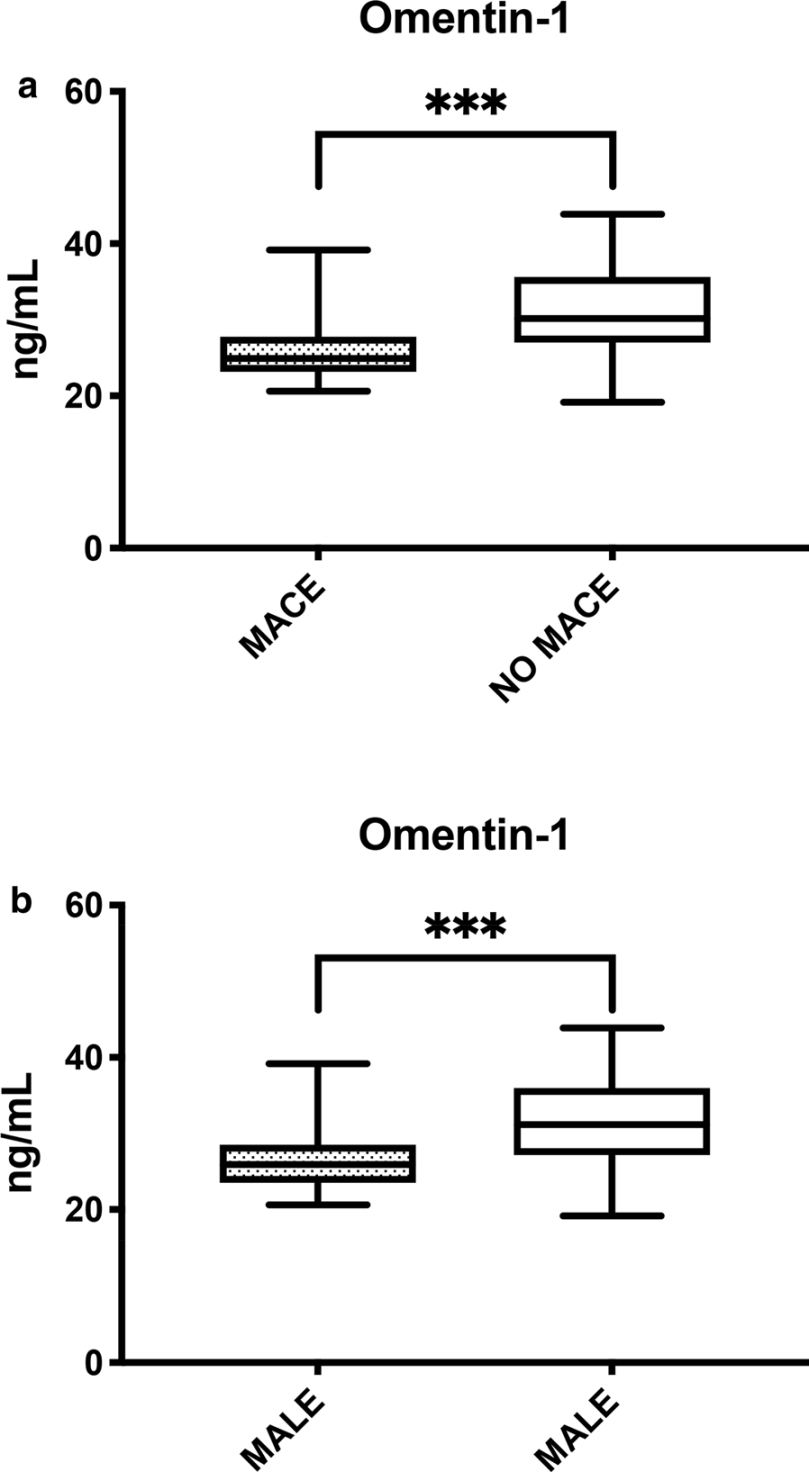
**a** Omentin-1 levels according to MACE. On the box plots, central lines represent the median, the length of the box represents the interquartile range and the lines extend to minimum and maximum values. ***P < 0.001. **b** Omentin-1 levels according to MALE. On the box plots, central lines represent the median, the length of the box represents the interquartile range and the lines extend to minimum and maximum values. ***P < 0.001.

**Fig. 3:**
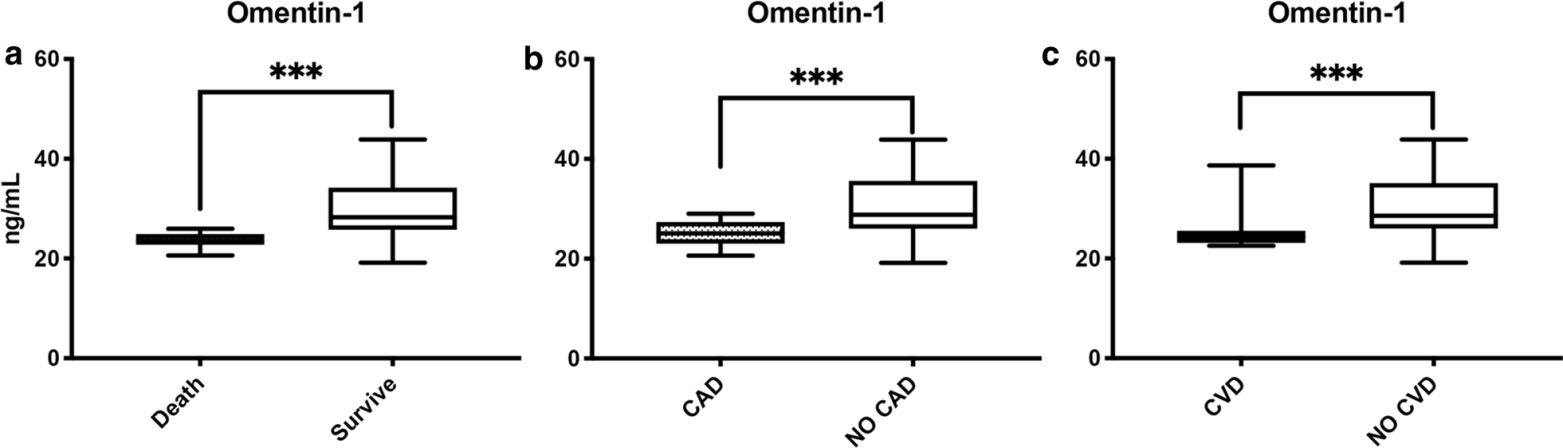
**a** Omentin-1 levels according to mortality. On the box plots, central lines represent the median, the length of the box represents the interquartile range and the lines extend to minimum and maximum values. ***P < 0.001. **b** Omentin-1 levels according to coronary artery disease (CAD) outcome. On the box plots, central lines represent the median, the length of the box represents the interquartile range and the lines extend to minimum and maximum values. ***P < 0.001. **c** Omentin-1 levels according to cerebrovascular disease (CVD) outcome. On the box plots, central lines represent the median, the length of the box represents the interquartile range and the lines extend to minimum and maximum values. ***P < 0.001.

During the 12-month follow-up period, 84 (40.6%) patients experienced MACE after LER. Complete clinical data from patients with MACE and without MACE are shown in Table 2. Considering baseline omentin-1 levels, patients with MACE had lower baseline serum levels (26.02 ± 4.05 vs 31.33 ± 5.29 ng/mL, p < 0.001) (Fig. 2a). Of these, 16 (7.7%) patients died (Fig. 3a) and had lower serum levels than survivors (23.71 ± 1.49 vs 29.63 ± 5.44 ng/mL, p < 0.001). Furthermore, 44 (21.3%) patients had a myocardial infarction and had lower omentin-1 levels than patients without CAD (25.22 ± 2.35 vs 30.24 ± 5.59 ng/mL, p < 0.001) (Fig. 3b). Moreover, 35 (16.9%) individuals had a stroke and lower levels than patients without CVD (25.06 ± 3.01 vs 30.01 ± 5.49 ng/mL, p < 0.001) (Fig. 3c).

**Table 2:** Demographic and clinical data of diabetic subjects with and without MACE

	NO MACE (n = 123)	MACE (n = 84)	p value
Age, years	75.244	74.584	0.606
Men:women, n	79:44	65:19	0.043
BMI, kg/m^2^	26.712	26.729	0.858
Diabetes duration, years	11.463	11.373	0.463
HBP, n (%)	81 (65.9)	63 (75.0)	0.160
HCHOL, n (%)	64 (52,0)	49 (58.3)	0.371
CAD, n (%)	55 (44.7)	40 (47.6)	0.681
CVD, n (%)	59 (48.0)	42 (52.0)	0.774
Current smokers, n (%)	61 (49.6)	66 (78.6)	< 0.001
Past smokers, n (%)	10 (8.1)	13 (15.5)	0.099
Never smoked, n (%)	52 (42.3)	5 (6.0)	< 0.001
Ankle Brachial Index	0.651	0.528	< 0.001
Total cholesterol, mg/dL	215.588	224.638	0.004
LDL cholesterol, mg/dL	107.097	120.691	< 0.001
Triglycerides, mg/dL	172.826	171.779	0.383
Glucose, mg/dL	128.114	127.823	0.832
HbA1C, %	8.832	8.974	0.151
eGFR mL/min/1.73 m^2^	73.096	72.459	0.67
Omentin-1 ng/mL	31.326	26.024	< 0.001

*BMI* Body Mass Index, *HBP* High Blood Pression, *HCHOL* Hypercholesterolemia, *CAD* Coronary Artery Disease, *CVD* Cerebrovascular Disease, *LDL* low-density lipoprotein, *eGFR* estimated glomerular filtration rate. Statistical test performed with Student’s t-test or with Chi square test, when appropriate.

**Fig. 4:**
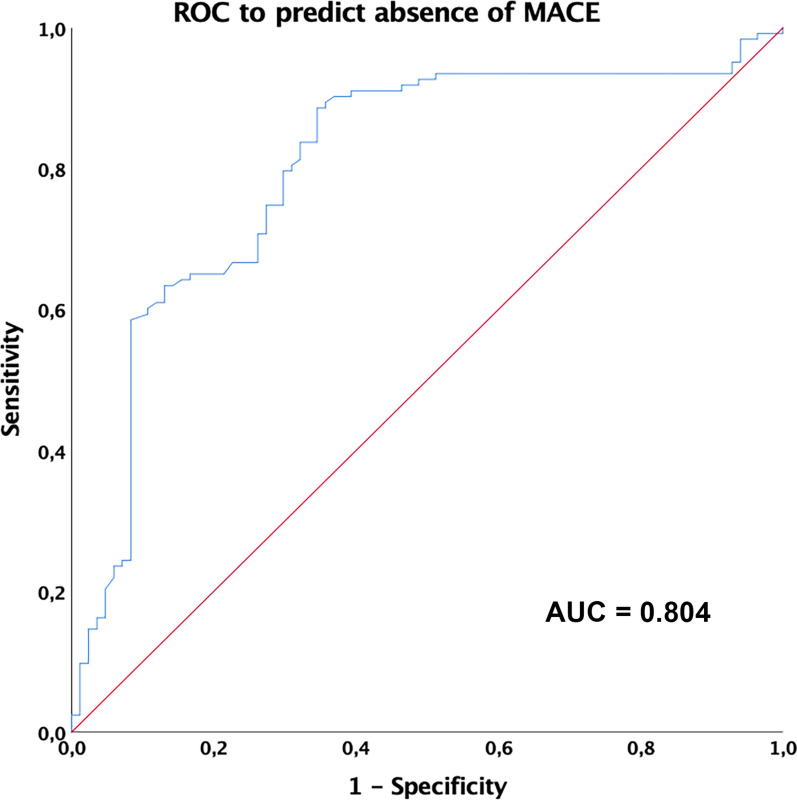
ROC curve analysis to predict absence of MACE related to omentin-1 levels in T2DM showing an area under the ROC curve of 0.804 (P < 0.001)

The area under the ROC curve to predict the incidence of MACE in T2DM patients, based on omentin-1 levels, was 0.80 (95% CI 0.74, 0.87) (Fig. 4). The best cut-off value of omentin-1 for prediction of the occurrence of MACE in our population was < 26.23 ng/mL (Sensitivity 79.7%, Specificity 70.2%).

**Fig. 5:**
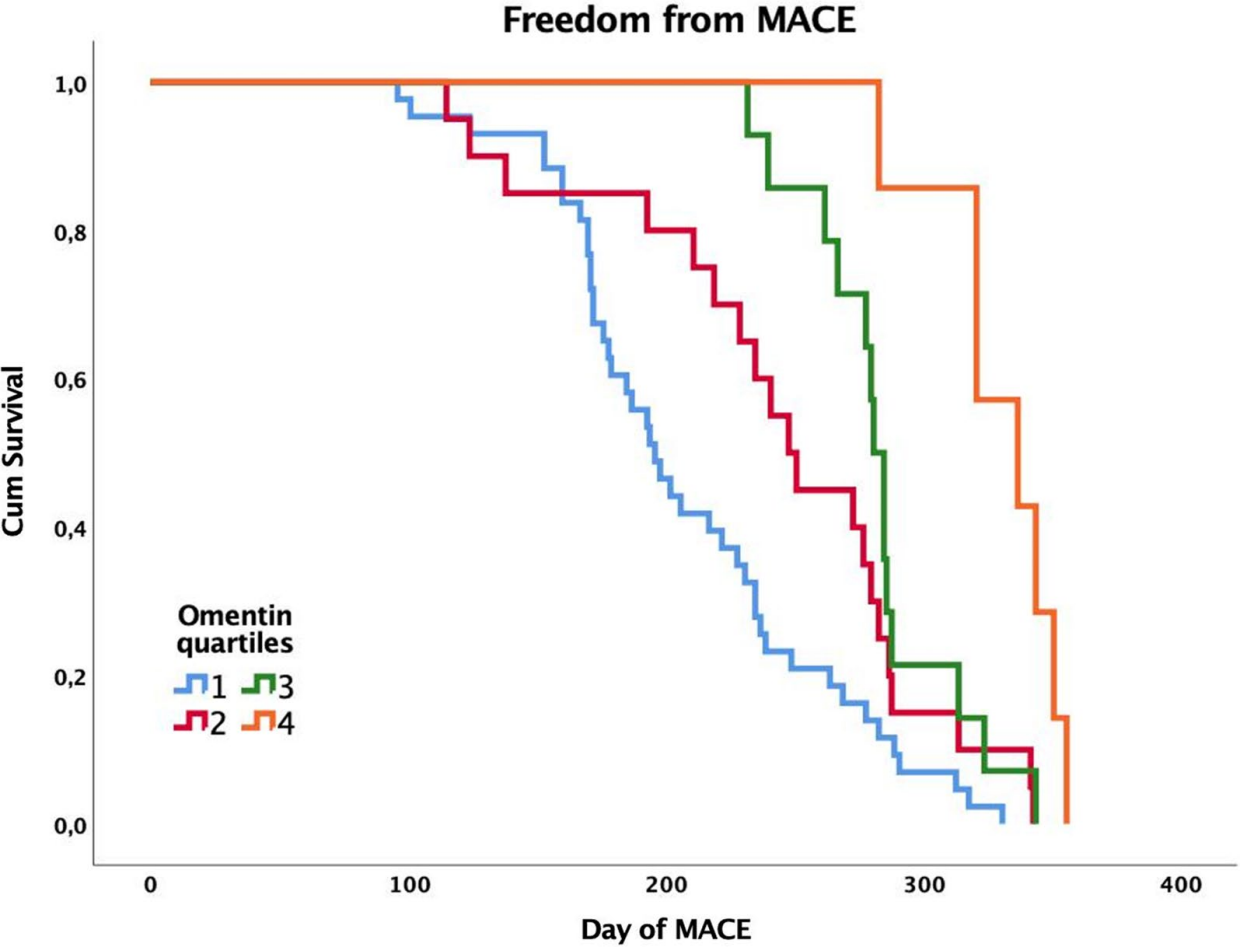
The freedom from MACE according to the quartiles of serum omentin-1 was estimated using the Kaplan–Meier method and compared using the Log-Rank test (P < 0.001). The quartiles of omentin-1 are listed in color code lines. Blue represents the first quartile, red the second, green the third, orange the fourth

Kaplan–Meier curves showing MACE-free survival according to omentin-1 quartiles are reported in Fig. 5. Omentin-1 quartile curves differed significantly (Log-Rank p < 0.001), and patients in the lowest quartile had the highest cumulative incidence of MACE.

The multivariate logistic regression analysis showed that, after adjustments for the cardiovascular risk factors age, male sex, BMI, smoking, hypertension, CAD, CVD, ABI, LDL-C, fasting plasma glucose and glycated hemoglobin, omentin-1 level was an independent determinant for MACE after LER in T2DM patients with PAD and with CLTI (p < 0.001, 95% CI −0.039, −0.014) (Table 3).

**Table 3:** Multivariable logistic regression for MACE

	Coef	St.Err	t-value	p-value	[95% Conf	Interval]	Sig
Age	−0.002	0.003	−0.74	0.457	−0.009	0.004	
Male sex	−0.044	0.070	−0.63	0.528	−0.182	0.093	
HBP	0.026	0.065	0.41	0.683	−0.101	0.154	
HCHOL	0.038	0.059	0.65	0.516	−0.078	0.155	
CAD	0.073	0.059	1.23	0.219	−0.044	0.190	
CVD	−0.011	0.060	−0.19	0.851	−0.130	0.107	
Current smokers	−0.040	0.097	−0.41	0.683	−0.232	0.152	
Past smokers	0.000						
Never smoked	−0.296	0.111	−2.67	0.008	−0.514	−0.077	***
ABI	−0.209	0.280	−0.75	0.456	−0.762	0.343	
LDL-C	0.006	0.002	2.59	0.010	0.001	0.011	**
FPG	0.003	0.003	0.87	0.384	−0.003	0.009	
HbA1C	0.046	0.042	1.07	0.284	−0.038	0.129	
Omentin-1	−0.025	0.007	−3.70	< 0.001	−0.038	−0.012	***
Constant	0.058	0.719	0.08	0.936	−1.360	1.476	
Mean dependent var	0.406		SD dependent var	0.492			
R-squared	0.349		Number of obs	207.000			
F-test	7.942		Prob > F	0.000			
Akaike crit. (AIC)	232.299		Bayesian crit. (BIC)	278.957			

***p < 0.01, **p < 0.05, *p < 0.1

Further multivariable logistic regression analyses demonstrated that omentin-1 level was an independent determinant for the incidence of CAD (p = 0.001, 95% CI −0.03, −0.007) and CVD (p < 0.001, 95% CI −0.032, −0.011) (Additional file 1: Tables S2 and S3). Only LDL-C remained an independent determinant for the incidence of death outcome (p < 0.001, 95% CI 0.003, 0.008) (Additional file 1: Table S1).

### Serum omentin-1 and risk of MALE at 12 months

During the 12-month follow-up, 96 (46.4%) patients had MALE following LER. Notably, we observed that omentin-1 levels were lower in patients that developed MALE than those who did not (26.67 ± 4.21 *vs* 31.34 ± 5.54 ng/mL, p < 0.001) (Fig. 2b). In the group of patients who had MALE, smoking status (p < 0.001), ABI (p < 0.001), LDL-C (p < 0.001) and Rutherford staging (p < 0.001) were significantly different from patients without MALE. The remaining clinical and laboratory characteristics are shown in Table 4.

**Table 4:** Demographic and clinical data of diabetic subjects with and without MALE

	NO MALE (n = 111)	MALE (n = 96)	p value
Age, years	74.901	75.062	0.898
Men:women, n	68:43	76:20	0.005
BMI, kg/m^2^	26.757	26.675	0.364
Diabetes duration, years	11.486	11.357	0.289
HBP, n (%)	73 (65,8)	71 (74.0)	0.201
HCHOL, n (%)	59 (53.2)	54 (56.3)	0.655
CAD, n (%)	52 (46.8)	43 (44.8)	0.767
CVD, n (%)	47 (42.3)	54 (56.3)	0.046
Current smokers, n (%)	59 (53.2)	68 (70.8)	0.009
Past smokers, n (%)	8 (7.2)	15 (15.6)	0.055
Never smoked, n (%)	44 (39.6)	13 (13.5)	< 0.001
Rutherford 4, n (%)	78 (70.3)	34 (35.4)	< 0.001
Rutherford 5, n (%)	33 (29.7)	62 (64.6)	< 0.001
Ankle Brachial Index	0.645	0.55	< 0.001
Total cholesterol, mg/dL	215.582	223.513	0.011
LDL cholesterol, mg/dL	107.898	118.067	< 0.001
Triglycerides, mg/dL	172.009	172.854	0.475
Glucose, mg/dL	127.611	128.441	0.539
HbA1C, %	8.919	8.857	0.519
eGFR mL/min/1.73 m^2^	72.489	73.24	0.611
Omentin-1 ng/mL	31.339	26.672	< 0.001

*BMI* Body Mass Index, *HBP* High Blood Pression, *HCHOL* Hypercholesterolemia, *CAD* Coronary Artery Disease, *CVD* Cerebrovascular Disease, *LDL* low-density lipoprotein, *eGFR* estimated glomerular filtration rate. Statistical test performed with Student’s t-test or with Chi square test, when appropriate.

The multivariate logistic regression analysis showed that, after adjustments for traditional cardiovascular risk factors, omentin-1 level was related to incidence of MALE after LER in T2DM patients with PAD and with CLTI (p < 0.001, 95% CI −0.041, −0.014) (Table 5).

**Table 5:** Multivariable logistic regression for MALE

	Coef	St.Err	t-value	p-value	[95% Conf	Interval]	Sig
Age	0.000	0.004	0.07	0.947	−0.007	0.007	
Male sex	0.037	0.075	0.49	0.623	−0.112	0.186	
HBP	0.034	0.070	0.49	0.625	−0.104	0.172	
HCHOL	−0.018	0.064	−0.28	0.779	−0.144	0.108	
CAD	−0.024	0.064	−0.37	0.714	−0.150	0.103	
CVD	0.126	0.065	1.93	0.055	−0.002	0.253	*
Current smokers	−0.089	0.105	−0.84	0.400	−0.297	0.119	
Past smokers	0.000						
Never smoked	−0.220	0.120	−1.83	0.068	−0.456	0.017	*
ABI	−0.208	0.303	−0.69	0.493	−0.806	0.390	
LDL-C	0.004	0.003	1.55	0.123	−0.001	0.009	
FPG	0.006	0.003	1.77	0.078	−0.001	0.012	*
HbA1C	−0.068	0.046	−1.49	0.137	−0.159	0.022	
Omentin-1	−0.026	0.007	−3.56	< 0.001	−0.041	−0.012	***
Constant	0.751	0.778	0.96	0.336	−0.783	2.285	
Mean dependent var	0.464		SD dependent var		0.500		
R-squared	0.261		Number of obs		207.000		
F-test	5.241		Prob > F		0.000		
Akaike crit. (AIC)	264.806		Bayesian crit. (BIC)		311.464		

*** p < 0.01, ** p < 0.05, * p < 0.1

## Discussion

T2DM patients with PAD have a higher risk of cardiovascular complications than T2DM patients without peripheral vascular involvement [2]. Despite multiple therapeutic approaches and multidisciplinary management, definite biomarkers, useful to stratify the risk for T2DM patients with PAD and CLTI are not available [25, 26]. Regarding follow-up after revascularization, even less clear evidence is available [27]. In fact, outcomes after LER vary widely among T2DM patients [4]. Despite similar baseline clinical characteristics—in terms of risk factor control and PAD severity—and the same endovascular approach, some patients do not encounter complications for years while other patients may have complications, even fatal, within a few months [27]. Several potential biomarkers have been proposed, some linked to patient's genetic characteristics [26, 28–30], many linked metabolic and inflammatory parameters [17, 26, 31–34]. Among the possible candidates, the pathways linked to adipose tissue represent a promising field of study. In particular, adipokines have been extensively studied and a strong relationship between these bioactive molecules and the mechanisms underlying vascular complications of T2DM [26]. In this scenario, omentin-1 could be an ideal biomarker.

### Study findings

The most important result of this study is that baseline omentin-1 levels correlate with the incidence of MACE during the period following LER in T2DM patients with PAD and with CLTI. Patients with lower omentin-1 levels had more myocardial infarctions, strokes and deaths. Amongst the explanations for our results, compelling evidence exists that demonstrates an inverse relationship between omentin-1 levels and some traditional risk factors for diabetic complications, including the amount and distribution of adipose tissue and glycemic control [35, 36]. In this sense, it is possible that reduced omentin-1 levels indirectly influenced the outcomes of our population, through the action of other risk factors. However, we did not observe differences in terms of BMI and glycemic balance in patients who had MACE compared to patients who did not. An additional possible explanation is that omentin-1 per se is a direct cardiovascular risk factor. This suggestion is in line with several previous evidences which demonstrate that omentin-1 levels are inversely correlated with the presence of CAD [8]. Indeed, omentin-1 levels are inversely related to the presence and extent of coronary artery atherosclerosis. Moreover, omentin-1 is inversely associated with intima-media thickness of the common carotid artery in high risk T2DM patients [37]. Furthermore, the serum levels of this protein are independently correlated with carotid atherosclerosis and the presence of carotid plaque, increasing the risk of stroke [38–40]. Accordingly, in our cohort, omentin-1 levels significantly decreased not only in the composite MACE outcome, but also in the three distinct adverse events—myocardial infarction, stroke and death—strengthening our findings further. An additional interesting result of our study, emerging from the ROC and Kaplan–Meier curve analysis, is that cut-off values exist identifying higher risk patients that could develop early vascular complication. This could allow physicians to design a personalized follow-up, based on omentin-1 levels before LER procedure.

The molecular mechanisms underlying the effects observed could be numerous. Omentin-1 can determine several important protective effects on the vascular system through nitric oxide, Akt and the AMP-activated protein kinase pathways [41]. In particular, by reducing endothelial dysfunction [42], oxidative stress and neointimal proliferation [43]. Omentin-1 can reduce the atherosclerotic process and the development of the myocardial infarction itself [44].

A further finding of this study is that baseline omentin-1 levels correlate with the incidence of MALE during the follow-up period. This result is of particular interest in T2DM patients frequently facing numerous complications associated to PAD during their clinical history. One of the possible explanations can be found in the initial stratification that demonstrates how omentin-1 levels correlate with PAD severity, in terms of ABI and Rutherford staging, which is in agreement with previous data [16]. The new and most interesting result is that the prospective nature of the study allows assigning a predictive significance to the initial omentin-1 levels. Additionally, multivariate analyzes confirm an effect independent of other vascular risk factors, such as smoking or increased LDL-C.

### Study limitations

Amongst the limitations of our study, the relatively small, single-center patient cohort and the impossibility to establish whether the results are replicable in a larger number of patients. Furthermore, it is unfeasible to determine whether omentin-1 levels play a direct role on the vascular risk of our cohort or if it is only an epiphenomenon. However, the aim of the investigation was to explore a risk stratification biomarker and, in this perspective, the study design was appropriate. A further limitation is that the association between statin therapy and omentin-1 levels, which may have affected the LDL-C target during follow-up, has not been evaluated. Furthermore, there are non-univocal data in the literature on the role of omentin-1 on cardiovascular risk factors. In fact, baseline omentin-1 levels have been associated with intima-media thickness in a diabetic population [45]. However, in this specific study, the presence of PAD was an exclusion criterion and it is not possible to make a comparison with our data. Moreover, in a large population, omentin-1 levels have been shown to be associated with stroke risk [12]. However, there were many differences in terms of race, BMI, metabolic status, follow-up and outcomes compared to our study. There are also data demonstrating an association between omentin-1 levels and cardiovascular events [46]. However, in this population only a quarter of the patients were diabetic and there were no differences related to the presence of diabetes. Furthermore, we have not found a significant association between MACE and previous CAD or CVD. Although several previous data confirm that an association between polyvascular atherosclerotic disease and the incidence of MACE does exist [47], we have not found this relationship in our diabetic population. This could partly be explained by the fact that the study population was exclusively composed of diabetic patients. In this scenario it is possible to hypothesize that the presence of T2DM in all patients is a more relevant risk factor than the previous CAD or CVD and that the influence of previous cardiovascular events in the incidence of MACE does not emerge. However, this conclusion is purely speculative and requires further confirmation on larger populations. Finally, an accurate analysis on the relationship between localization of initial arterial stenosis, restenosis and MALE has not been performed. The restrictive selection criteria, also excluding 17 patients where revascularization failed, reduced bias.

## Conclusion

In conclusion, this study demonstrated that baseline levels of omentin-1 correlate with the onset of vascular complications, in particular MACE and MALE, after LER in T2DM patients with PAD and CLTI. Further investigations on a larger sample of patients are needed to confirm our findings.

## Supplementary information

**Additional file 1: Table S1.** Multivariable logistic regression for Death. ** Table S2.** Multivariable logistic regression for CAD. **Table S3.** Multivariable logistic regression for CVD.

## Data Availability

The datasets generated during the current study are available from the corresponding author on reasonable request.

## Abbreviations

ABI: Ankle/brachial index
BMI: Body mass index
CAD: Coronary artery disease
CLTI: Chronic limb-threatening ischemia
CVD: Cerebrovascular disease
eGFR: Estimated glomerular filtration rate
LDL-C: Low-density lipoprotein cholesterol
LER: Lower limb revascularization
MACE: Major adverse cardiovascular events
MALE: Major adverse limb events
PAD: Peripheral artery disease
ROC: Receiver operating characteristics
T2DM: Type 2 diabetes mellitus
US: Ultrasound
WIfI: Wound, ischemia, foot infection
