# Supplementary material for: Association between omentin-1 and major cardiovascular events after lower extremity endovascular revascularization in diabetic patients: a prospective cohort study
Source: Cardiovasc Diabetol. 2020 Oct 7;19:170. doi: 10.1186/s12933-020-01151-z (PMC7542958; doi:10.1186/s12933-020-01151-z)
Supplement: Supplementary file 1 — Additional file 1: Table S1. Multivariable logistic regression for Death. Table S2. Multivariable logistic regression for CAD. Table S3. Multivariable logistic regression for CVD. [file 12933_2020_1151_MOESM1_ESM.docx]

**Table S1. Multivariable logistic regression for Death**

|  | Coef. | | St.Err. | t-value | | p-value | [95% Conf | | Interval] | | Sig |
| --- | --- | --- | --- | --- | --- | --- | --- | --- | --- | --- | --- |
| Age | 0.003 | | 0.002 | 1.47 | | 0.144 | -0.001 | | 0.007 | |  |
| Male sex | -0.004 | | 0.042 | -0.10 | | 0.919 | -0.086 | | 0.078 | |  |
| HBP | 0.034 | | 0.039 | 0.89 | | 0.376 | -0.042 | | 0.11 | |  |
| HCHOL | 0.064 | | 0.035 | 1.81 | | 0.071 | -0.006 | | 0.134 | | * |
| CAD | -0.01 | | 0.035 | -0.29 | | 0.776 | -0.08 | | 0.06 | |  |
| CVD | 0.028 | | 0.036 | 0.78 | | 0.439 | -0.043 | | 0.098 | |  |
| Current smokers | -0.009 | | 0.058 | -0.15 | | 0.879 | -0.123 | | 0.105 | |  |
| Past smokers | 0 | | . | . | | . | . | | . | |  |
| Never smoked | -0.016 | | 0.064 | -0.25 | | 0.806 | -0.143 | | 0.111 | |  |
| LDL-C | 0.006 | | 0.001 | 4.29 | | <0.001 | 0.003 | | 0.008 | | *** |
| FPG | 0.003 | | 0.002 | 1.47 | | 0.143 | -0.001 | | 0.006 | |  |
| HbA1C | -0.017 | | 0.025 | -0.67 | | 0.503 | -0.067 | | 0.033 | |  |
| Omentin-1 | -0.006 | | 0.004 | -1.65 | | 0.101 | -0.014 | | 0.001 | |  |
| Constant | -0.844 | | 0.414 | -2.04 | | 0.043 | -1.661 | | -.027 | | ** |
|  | | | | | | | | | | | |
| Mean dependent var | | 0.077 | | | SD dependent var | | | 0.268 | |  |  |
| R-squared | | 0.210 | | | Number of obs | | | 207.000 | |  |  |
| F-test | | 4.293 | | | Prob > F | | | 0.000 | |  |  |
| Akaike crit. (AIC) | | 18.096 | | | Bayesian crit. (BIC) | | | 61.421 | |  |  |
| **** p<.01, ** p<.05, * p<.1* | | | | | | | | | | | |
|  | | | | | | | | | | | |

**Table S2. Multivariable logistic regression for CAD**

|  | Coef. | | St.Err. | t-value | | p-value | [95% Conf | | Interval] | | Sig |
| --- | --- | --- | --- | --- | --- | --- | --- | --- | --- | --- | --- |
| Age | 0.006 | | 0.003 | 1.91 | | 0.058 | <0.001 | | 0.011 | | * |
| Male sex | 0.002 | | 0.063 | 0.03 | | 0.98 | -0.122 | | 0.125 | |  |
| HBP | 0.071 | | 0.058 | 1.22 | | 0.224 | -0.044 | | 0.186 | |  |
| HCHOL | 0.053 | | 0.053 | 0.99 | | 0.324 | -0.052 | | 0.157 | |  |
| CAD | -0.002 | | 0.053 | -0.03 | | 0.973 | -0.107 | | 0.103 | |  |
| CVD | 0.031 | | 0.054 | 0.58 | | 0.56 | -0.075 | | 0.138 | |  |
| Current smokers | -0.124 | | 0.087 | -1.43 | | 0.155 | -0.296 | | 0.048 | |  |
| Past smokers | 0 | | . | . | | . | . | | . | |  |
| Never smoked | -0.249 | | 0.097 | -2.57 | | 0.011 | -0.44 | | -0.058 | | ** |
| LDL-C | 0.004 | | 0.002 | 1.85 | | 0.066 | <0.001 | | 0.008 | | * |
| FPG | 0.006 | | 0.003 | 2.18 | | 0.03 | 0.001 | | 0.011 | | ** |
| HbA1C | -0.001 | | 0.038 | -0.03 | | 0.979 | -0.076 | | 0.074 | |  |
| Omentin-1 | -0.019 | | 0.006 | -3.24 | | 0.001 | -0.03 | | -0.007 | | *** |
| Constant | -0.785 | | 0.623 | -1.26 | | 0.209 | -2.015 | | 0.444 | |  |
|  | | | | | | | | | | | |
| Mean dependent var | | 0.213 | | | SD dependent var | | | 0.410 | |  |  |
| R-squared | | 0.238 | | | Number of obs | | | 207.000 | |  |  |
| F-test | | 5.036 | | | Prob > F | | | 0.000 | |  |  |
| Akaike crit. (AIC) | | 187.294 | | | Bayesian crit. (BIC) | | | 230.619 | |  |  |
| **** p<.01, ** p<.05, * p<.1* | | | | | | | | | | | |
|  | | | | | | | | | | | |

**Table S3. Multivariable logistic regression for CVD**

|  | Coef. | | St.Err. | t-value | | p-value | [95% Conf | | Interval] | | Sig |
| --- | --- | --- | --- | --- | --- | --- | --- | --- | --- | --- | --- |
| Age | -0.002 | | 0.003 | -0.68 | | 0.496 | -0.007 | | 0.004 | |  |
| Male sex | -0.114 | | 0.059 | -1.94 | | 0.054 | -0.231 | | 0.002 | | * |
| HBP | 0.033 | | 0.055 | 0.60 | | 0.547 | -0.075 | | 0.141 | |  |
| HCHOL | 0.078 | | 0.05 | 1.56 | | 0.121 | -0.021 | | 0.177 | |  |
| CAD | -0.028 | | 0.05 | -0.55 | | 0.584 | -0.126 | | 0.071 | |  |
| CVD | 0.08 | | 0.051 | 1.57 | | 0.119 | -0.021 | | 0.18 | |  |
| Current smokers | 0.102 | | 0.082 | 1.24 | | 0.217 | -0.06 | | 0.264 | |  |
| Past smokers | 0 | | . | . | | . | . | | . | |  |
| Never smoked | 0.039 | | 0.091 | 0.43 | | 0.67 | -0.141 | | 0.219 | |  |
| LDL-C | 0.003 | | 0.002 | 1.70 | | 0.091 | -0.001 | | 0.007 | | * |
| FPG | 0.001 | | 0.003 | 0.21 | | 0.836 | -0.005 | | 0.006 | |  |
| HbA1C | -0.026 | | 0.036 | -0.73 | | 0.467 | -0.097 | | 0.044 | |  |
| Omentin-1 | -0.021 | | 0.005 | -3.92 | | <0.001 | -0.032 | | -0.011 | | *** |
| Constant | 0.653 | | 0.588 | 1.11 | | 0.268 | -0.506 | | 1.812 | |  |
|  | | | | | | | | | | | |
| Mean dependent var | | 0.169 | | | SD dependent var | | | 0.376 | |  |  |
| R-squared | | 0.193 | | | Number of obs | | | 207.000 | |  |  |
| F-test | | 3.857 | | | Prob > F | | | 0.000 | |  |  |
| Akaike crit. (AIC) | | 162.891 | | | Bayesian crit. (BIC) | | | 206.216 | |  |  |
| **** p<.01, ** p<.05, * p<.1* | | | | | | | | | | | |
|  | | | | | | | | | | | |
